# Diabetes Mellitus in the Middle-Aged and Elderly Population (>45 Years) and Its Association With Pancreatic Cancer: An Updated Review

**DOI:** 10.7759/cureus.8884

**Published:** 2020-06-28

**Authors:** Thanmai Kaleru, Varun K Vankeshwaram, Ankush Maheshwary, Divya Mohite, Safeera Khan

**Affiliations:** 1 Internal Medicine, California Institute of Behavioral Neurosciences and Psychology, Fairfield, USA; 2 Medicine, Zaporozhye State Medical University, Zaporozhye, UKR; 3 Neurology, California Institute of Behavioral Neurosciences and Psychology, Fairfield, USA; 4 Medicine, Government Medical College, Amritsar, IND

**Keywords:** diabetes mellitus in elderly, new-onset diabetes, pancreatic cancer

## Abstract

Diabetes mellitus (DM) and pancreatic cancer (PC) in the elderly are widely considered to be interrelated. New-onset diabetes (NOD) patients are considered a high-risk group for the development of PC within three years of diagnosis. We reviewed the literature to determine the pathophysiological association between DM and PC, which can help in the development of screening tests for early PC diagnosis in the elderly with NOD. We also studied the potential associations between them after pancreaticoduodenectomy (PD) or pancreatic resection. We collected studies published in the last five years in PubMed that are relevant to DM and PC in the elderly. We mainly focused on the pathophysiology and intracellular mechanisms involved between NOD and PC. We illustrated the clinical signs and immunological and metabolic biomarkers that can be used to diagnose early PC in the elderly with NOD. In the 34 studies we reviewed, five showed that long-term diabetes mellitus (LTDM) increases the risk of PC. Six studies showed that NOD in the elderly is an early sign of PC. Fourteen studies proposed that clinical signs and biomarker levels should be used to determine the high-risk risk group for PC among NOD patients. Six studies reported that NOD is associated with the worst outcomes postoperatively, and three studies showed that patients developed DM after pancreatic resection. LTDM is considered an independent risk factor for PC development in the elderly. NOD is a consequence and maybe the only early presenting sign of PC. Screening protocols and tests should be used in clinical practice to determine the proportion of NOD patients who should undergo further testing for early diagnosis of PC. DM and PC are also co-related postoperatively and patients should be monitored for impaired glucose levels, overall survival, and mortality.

## Introduction and background

Diabetes Mellitus (DM) is a complex metabolic disorder with characteristic hyperglycemia that arises either due to defective insulin secretion by pancreatic islets or due to insulin resistance of peripheral tissues or deregulated hepatic glucose production. Diagnostic criteria is defined as two consecutive readings of fasting blood glucose (FBG) of >126 mg/dl; two-hour post-glucose level of >200 mg/dl (oral glucose tolerance test); random blood glucose level of >200 mg/dl; or glycated hemoglobin (HbA1c) level of >6.5% [1]. The global prevalence of diabetes as reported by the International Diabetes Federation in 2019 was 9.3%, and it is estimated to rise to 10.2% by 2030 [2].

DM manifests as either type 1 (due to beta cell destruction, leading to absolute insulin deficiency) or type 2 (predominantly due to insulin resistance with relative insulin deficiency or insulin secretory defect). Other, less common types of diabetes are as follows: those associated with genetic defects of beta cells or insulin action, drug and chemical-induced diabetes, endocrinopathies, and disease of the exocrine pancreas (type 3c diabetes). In this review, we considered new-onset diabetes (NOD) in the last two to three years as a diagnosis of DM in patients with an FBG of >126 mg/dL or HbA1c of >6.5% without a known history of diabetes. Long-term diabetes mellitus (LTDM) was defined as a diagnosis of DM made for more than three years. Typically, type 1 diabetes is diagnosed among younger patients. On the other hand, type 2 DM caused by insulin resistance is the most common type seen in older populations. In this article, DM is referred to as type 2 diabetes by default. The average range of peak age of diagnosis of type 2 DM is considered to be between 45-60 years [3]. Another study published by the American Diabetes Association (ADA) states that type 3c diabetes (diabetes following pancreatic disease) often misdiagnosed as type 2 DM is associated with poor glycemic control and has a higher incidence than type 1 DM, with a peak age of 59 years [4]. According to the Centers for Disease Control and Prevention (CDC) reports, the incidence of any type of diabetes increases with age and tends to stem off after the age of 65 years [2]. Long-term diabetes is prevalent in the elderly and is a risk factor for all malignancies, including pancreatic cancer (PC) [5]. There are many risk factors (smoking, age, alcohol, chronic pancreatitis, diabetes, obesity, genetic, and other cancer syndromes) contributing to the development of PC [6]. Many studies have documented a positive association between LTDM and PC [5,7,8,9]. This can be explained by the risk factors (age, obesity, genetic factors, and lifestyle changes) or pathophysiology (impaired action of the insulin) shared by the two diseases. NOD in the elderly is considered to be highly associated with PC, with reported evidence of nearly 40% of patients diagnosed with NOD developing PC within three years of diagnosis [10,11].

PC is less prevalent, yet it is one of the deadly malignancies with high death rates [12]. According to 2019 cancer statistics, it is the tenth most common type of cancer among women and ninth in men, but the fourth leading cause of cancer-related deaths among all the cancers in the US [13]. Sporadic PC is more prevalent in individuals who are more than 50 years of age with NOD [14,15]. Pancreatic adenocarcinoma (PAC), arising from the pancreatic ductal cells, is the most common type of PC and accounts for 90% of all PC cases. In this review, we have referred to PAC as PC. Most patients with PC are asymptomatic or have vague and unexplained symptoms like pain, weight loss, jaundice, loss of appetite, nausea, pancreatitis, and new onset of diabetes [16]. Imaging tests (CT, MRI, and endoscopic ultrasound) are the main screening modalities used for the diagnosis of PC. However, due to the low incidence and the early asymptomatic phase of the tumor, population-based screening is not practiced in the clinical setting. Hence, the majority (85%) of the patients at the time of diagnosis are in their advanced stages and are not eligible for surgical resection [14,17]. Thus, early screenings have a significant impact on the prognosis of this aggressive tumor. We performed a literature review of the articles explaining the association between the two diseases and reported the potential pathophysiological and intracellular mechanisms involved.

Early and effective detection programs in the past decade have improved the treatment options and prognosis and reduced the mortality rates of other cancers [15]. However, in the case of PC, the advances have been minimal due to the uncertainty regarding the proportion of patients to be tested. Elderly patients with NOD represent a high-risk group and should be potentially screened for early diagnosis of PC. Proposed clinical models and interventional strategies may help in early diagnosis [15]. However, there is still a lack of clarity regarding standard criteria and diagnostic tests that differentiate NOD from NOD-PC (new-onset diabetes with pancreatic cancer). In this review, we discuss the various clinical signs and immunological and metabolic biomarkers that have proven useful in distinguishing the subpopulation of NOD-PC from NOD in the elderly. We also discuss the clinical outcomes in terms of glucose levels and prognosis of PC post-surgery.

## Review

Methods

**Figure 1:**
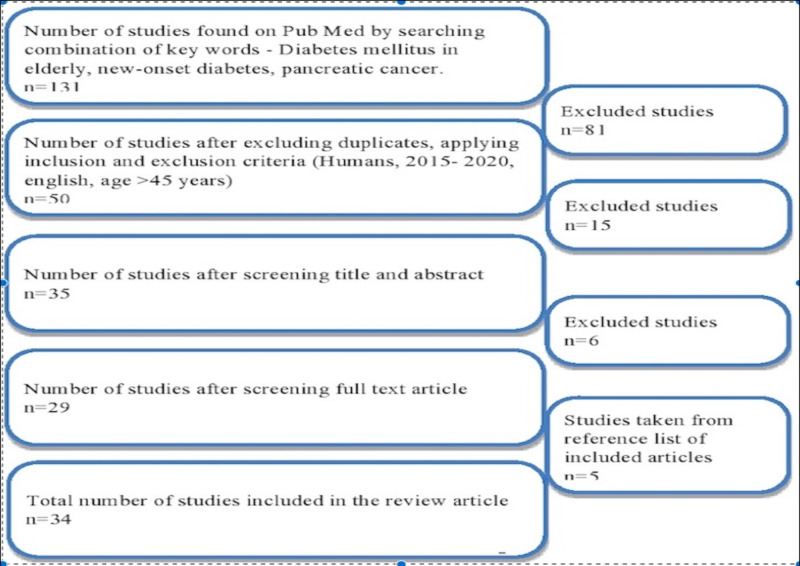
PRISMA diagram of the studies included PRISMA: preferred reporting items for systematic review and meta-analysis

We followed the preferred reporting items for systematic review and meta-analysis (PRISMA) guidelines (Figure 1) and relied mainly on PubMed to search for articles that are relevant to diabetes and PC and explain the association between these two diseases. We conducted the literature search by using the combination of keywords “diabetes mellitus in elderly,” “new-onset diabetes,” and “pancreatic cancer.”

We included peer-reviewed studies that are relevant to the topic. All the studies that are included were published in the English language within the last five years before the search date (2015-2020). We did not focus on any specific geographical location for the study search. However, we limited our search to the middle-aged and elderly population (>45 years). Our study results included both the abstract and full-text articles and did not include any form of grey literature.

We focused on the potential pathophysiology and intracellular mechanisms involved between NOD and PAC. We excluded the mechanisms and associations involved in other types of PC. We particularly focused on changes in the clinical signs and immunological and metabolic biomarkers that can be used to diagnose early PC in the elderly with NOD.

All data were collected in an ethical and legal manner. Quality appraisal of the studies included was done using the Cochrane risk-of-bias tool for randomized trials, Newcastle-Ottawa Scale for observational studies, and the assessment of multiple systematic reviews (AMSTAR) checklists for systematic reviews/meta-analyses.

Results

By following the search criteria mentioned in the methods section, we collected a total of 50 articles that are relevant to NOD and PC. We further filtered the relevant articles by reading the title, abstract, and full text to a final number of 34 articles included in this review. Among them, six are randomized control trials, 19 are observational studies, one is a systemic review, and three are meta-analyses. The remaining three are clinical guidelines and other publications. All of the studies included are peer-reviewed.

Among the included articles, four studies explained that LTDM is an independent risk factor for PC [5,7,8,9]. Seven studies showed that NOD precedes the onset of PC [9-12,18-20], and 13 studies proposed as to how NOD with certain clinical factors and screening tests can be used as a predictor for PC screening [1,7,12,14,16,18,19,21-27]. Seven studies mentioned the relation between PC and DM after pancreatic surgery [28-34]. Details of a few of the important studies are summarized in Table 1.

**Table 1:** Description of selected studies included in the review LTDM: long-term diabetes mellitus; PC: pancreatic cancer; NOD: new-onset diabetes mellitus; END-PAC: enriching new-onset diabetes for pancreatic cancer; CA 19-9: carbohydrate antigen 19-9; NOD-PC: new-onset diabetes with pancreatic cancer; RR: relative risk; OR: odds ratio; HbA1c: glycated hemoglobin

Author name and year of publication	Type of study and number of subjects included (n)	Study purpose	Results	Conclusion
Sharma et al., 2018 [14]	Observational cohort study; n=1,561	To develop a model to determine the risk of PC in NOD patients	Patients with an END-PAC score of zero has an extremely low risk of PC. A score of >3 identified 75% of patients with six months' early diagnosis of PC	Change in weight, blood glucose, and age at onset of diabetes can be used as a model (END-PAC) to contribute to early detection of PC
Choe et al., 2018 [16]	Observational study; n=5,111	To assess the utility of CA 19-9 as a screening indicator of PC with asymptomatic patients with NOD	PC is detected in 3.8% of patients with normal bilirubin levels and high CA 19-9 levels and 0.3% with normal CA 19-9 levels	CA 19-9 can be a useful biomarker of PC in patients after diagnosis of NOD
Mueller et al., 2019 [21]	Case-control study	To evaluate blood glucose levels and weight change as predictors of PC in NOD	Weight loss of >15% is associated with an OR of 4.56 compared to a stable weight, and HbA1c of <5.1 mmol/L before two to three years of cancer has OR of 2.42 compared to HbA1c of >6.3 mmol/L	Normal weight and blood glucose levels before diabetes onset may be predictive of NOD-PC
Lee et al., 2018 [29]	Observational study	To analyze clinical outcomes of PC with NOD	Overall survival of patients with NOD was worse than non-DM 22 vs. 33 months	NOD represents aggressive tumor biology of PC
Song et al., 2015 [5]	Meta-analysis	To determine if LTDM is an independent risk factor for PC	RR of PC with a duration of diabetes of >2 years, >5 years, and >10 years was 1.64, 1.58, 1.50 respectively	LTDM (>2 years) is associated with an increased risk of PC but negatively correlated with the duration

Discussion

**Figure 2:**
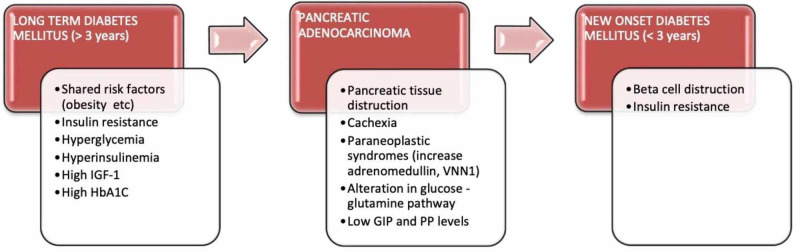
Pathophysiology of the association between diabetes mellitus and pancreatic cancer IGF-1: insulin-like growth factor 1; HbA1c: glycated hemoglobin; VNN1: vanin-1; GIP: glucose-dependent insulinotropic peptide; PP: pancreatic polypeptide

There is a bidirectional relationship between DM and PC. Overall, DM is associated with a two-fold increased risk of PC. When it is further stratified according to the duration of onset, NOD (<3 years) is associated with a 1.5-1.7 fold increase in the risk of PC [12,5]. LTDM is considered an independent risk factor for PC and, on the other hand, NOD is considered an early manifestation. The pathophysiological mechanisms involved in this relation are complicated and are explained in Figure 2. To elucidate the underlying pathophysiology and propose a feasible PC screening strategy for early diagnosis among the high-risk groups, we interpreted and analyzed various studies that explained the potential mechanisms, screening models, and tests.

Pathophysiology of the Association Between PC and DM

A meta-analysis published in 2015 showed that diabetes lasting more than two years is considered as an independent risk factor for PC [5]. The likely mechanism explained for this increased risk of carcinogenesis is related to the simultaneous increase in both the insulin-like growth factor-1 (IGF-1) levels and insulin signaling pathways, which causes hyperglycemia, insulin resistance, and hyperinsulinemia. Increased IGF-1 levels cause tumor pathogenesis by inducing the proliferation and inhibition of apoptosis in the target tissues. Hyperglycemia can also cause oxidative stress in the tissues by increasing the reactive oxygen species. However, it is not clear if it is the above pathogenesis or the shared risk factors like obesity that play a major role in an increased incidence of PC in patients with DM. Another pooled analysis conducted by McWilliams et al. in 2016 tested the risk factors for very early (<45 years age) and early (age less than 60 years) onset of PC and proposed that LTDM appeared to be more effective in the development of PC in older ages (>45 years) compared to younger patients (<45 years) [9]. However, the difference in the risk between the two age groups was not fully explained. High HbA1c levels at the onset of diabetes further correlate with a higher incidence of PC [8]. However, this correlation was only explained in patients with NOD (<3 years). On the other hand, complications of LTDM like kidney failure and high serum creatine are inversely related to PC development [7]. This could be due to the high mortality rate in diabetics with these complications. There is limited literature available on the role of anti-diabetic medications in the development of PC. However, some studies suggested that the use of metformin is associated with minimal risk or protective against PC, and the use of insulin is associated with the highest risk [8]. From all these studies, it is evident that prolonged increased levels of glucose and insulin are the main components that lead to carcinogenesis.

Significantly, 73% of patients with PC were assessed to have recently diagnosed with DM or pre-diabetes within two years retrospectively [10]. This type of cancer-related NOD is highly associated with the elderly because the prevalence of PC increases and NOD decreases after the age of 65 years [9]. Investigation of underlying mechanisms of PC-induced NOD might help to come up with the potential strategies for early screening, diagnosis, and treatment. Two potential phenomena explained previously are progressive pancreatic tissue destruction by tumor growth and cachexia, and the other is by the development of insulin resistance as one of the paraneoplastic syndromes caused by tumor secreting products [11]. However, the size of the tumor is not directly proportional to the blood glucose levels. It is further explained by high glucose levels in the early stages when the size of the tumor is very small for detection. Another study published earlier argued that the loss of beta-cell function through hormonal mechanisms plays a major role compared to insulin resistance in the development of NOD in PC [18]. It suggested that increased expression of proteases results in decreased levels of selective gastrointestinal hormones like glucose-dependent insulinotropic peptide (GIP), pancreatic polypeptide (PP), and a further decrease in insulin secretion from beta cells. High adrenomedullin levels are found in NOD-PC compared to NOD alone [12]. There is also overexpression of vanin-1 (VNN1), and its downstream metabolic products are observed in PC [19]. These were found to exacerbate paraneoplastic diabetes through the synergetic damage to the islet cells by triggering oxidative stress within the pancreatic microenvironment. Alterations in the circulating levels of several metabolites of amino acids, glucose and glutamine pathways, bile acid, and sphingolipids have been observed in NOD-PC [20]. Detection of these biomarker levels can help to stratify the subpopulation of high-risk patients from the NOD cohort that could be referred for screening with imaging. However, these were observational findings, and further interventional studies are required to validate the significance of using them in PC screening. A different pathway of cancer-genesis without affecting the glucose metabolism is explained by high microRNA (mRNA) levels; mainly, a significant increase in mRNA-200 and mRNA-192 in severe and progressive PC is observed [10]. Measuring their levels might help in cases of susceptible high-risk patients with normal glucose levels. 

Screening Strategies for Early Diagnosis of PC in Patients with NOD

NOD could be the earliest sign of PC and patients with NOD are considered as a high-risk group for early-stage, asymptomatic cancer. A sudden increase in the blood glucose levels in diabetics who were well controlled previously may also be a sign of PC. However, screening all the elderly NOD patients with imaging tests for PC is challenging in the clinical practice and not cost-effective. Several clinical strategies have been proposed that might be helpful as an initial filter to stratify individuals that can be referred for further screening. A retrospective, population-based cohort study conducted in the United Kingdom assessed clinical parameters like age, BMI, change in the BMI, HbA1c levels, anti-diabetic medications, and other factors and developed the risk model that stated that only 6.19% would undergo and benefit from the definitive screening of PC if the risk threshold for PC within three years was set at 1% [7]. However, this model also included the younger age groups (>35) compared to the individuals that are the focus of the current review article (>45). It also has to be validated externally before it can be considered in clinical use. Another prospective observational cohort study initiated by the Consortium for the Study of Chronic Pancreatitis, Diabetes, and Pancreatic Cancer (CPDPC) proposed a new approach (define, enrich, find) to define and enrich the high-risk group of PC and find the lesion in the high-risk cohort [1]. The use of enriching new-onset diabetes for pancreatic cancer (END-PAC) score to stratify subjects of >50 years into high, intermediate, and low-risk groups can also be an effective tool for clinicians to proceed for further screening [14]. Older patients with weight loss (low BMI) and a rapid increase in glucose levels in a short duration are predictive of PC and could be the target population for further screening [21]. Other useful differentiating factors predicted to be tested to differentiate NOD from NOD-PC are BMI, age of onset, hepatitis B virus (HBV), total bilirubin (TBIL), alanine aminotransferase (ALT), creatinine (Cr), apolipoprotein-A1 (APO-A1), and white blood cells (WBC) [22]. Fatigue and depression caused by elevated cytokine production, interleukin 6 (IL-6), along with severe weight loss (>10%) and NOD can be early paraneoplastic signs and potential precursors for PC [23]. A prediction model of medicare enrollees provided some information about the risk factors that could be used to test for emergent diagnosis of PC. However, it was not applicable for population screening since they have excluded the data for three months before the development of PC [24]. All the above studies are to be validated further by conducting population-based prospective interventional studies to find out the best strategy. However, clinical implementation of these strategies may reduce the dilemma regarding PC screening in NOD patients.

Many other studies have discussed the potential screening tests for early diagnosis of PC. Carbohydrate antigen (CA) 19-9 level excreted by cancer cells is a well-established indicator of PC with NOD compared to asymptomatic PC patients. However, it has a high false-positive rate as it is increased in any condition that leads to inflammation of the pancreas. A study has recommended assessing the CA 19-9 and liver function tests (LFT) in NOD patients seen in clinical settings [16]. An earlier study revealed that CA 19-9 levels are not significant in screening PC in NOD as both positive predictive value (PPV) and sensitivity were zero and had a false positive rate (FPR) of 9% [25]. But this was tested in a small number of cases and cannot be applied to the population. The same study also mentioned that CT was a reliable screening method compared to the ultrasound. However, the risk of radiation exposure should be weighed over its benefits, and other easy, noninvasive screening tests should be used in the first stages of clinical encounter. High levels of bilirubin are considered a more effective test for tumors arising from the head of the pancreas as it correlates with PC development even with CA 19-9 levels being normal (<37 U/ml). Therefore, CA 19-9 could be considered as a better screening test with normal bilirubin levels, which helps in detecting PC arising from the tail of the pancreas. A later study also emphasizes that CA 19-9 could be used as a cost-effective test in screening early PC lesions in the first two years of NOD development, which are too small to be detected on imaging studies [26]. This helps to alert the clinicians for regular monitoring of PC in NOD patients even with negative imaging results. Alterations in levels of metabolites of primary bile acid synthesis, sphingolipid, and amino acid metabolism are also observed in cases with NOD-PC compared to controls of NOD patients [20]. Studies have shown that tumor-secreted products like adrenomedullin, mRNA panel, VNN1 and its downstream molecules, and gastrointestinal hormones like GIP and PP can be used as the biomarkers potentially useful in distinguishing type 2 DM from DM with PC [12,27,19,18]. However, a more comprehensive panel of the tumor-secreted diabetogenic factors that result in the paraneoplastic development of NOD should be used to implement screening along with clinical characteristics and CA 19-9 levels. The clinical and biological factors that help to distinguish between NOD-PC and NOD are briefly explained below (Table 2).

**Table 2:** Distinguishing clinical and biological factors between NOD-PC and NOD NOD: new-onset diabetes; NOD-PC: new-onset diabetes with pancreatic cancer; BMI: body mass index; VNN1: vanin-1; GIP: glucose-dependent insulinotropic peptide; PP: pancreatic polypeptide

Clinical signs/biomarkers	NOD with pancreatic cancer (NOD-PC)	NOD without pancreatic cancer (NOD)
Patient age	>65 years	<45 years
Change in weight/BMI	Rapid weight loss/low BMI	A gradual loss of weight/high BMI
Change in glucose levels	A rapid change in a short period of time, higher HbA1c, higher glucose levels	A gradual change in a longer duration of time, lower HbA1c, lower glucose levels
Adrenomedullin	Increased	Normal
VNN1 and metabolites	Increased	Normal
Micro RNAs	Increased	Normal
Metabolomics	Increased	Normal
GIP, PP	Decreased	Normal

Association Between DM and PC Regarding Prognosis After Pancreatic Resection (Pancreatoduodenectomy)

Surgical resection or pancreatoduodenectomy (PD) is the most effective treatment for PC compared to chemotherapy or other treatment options. Even though the prognosis after treatment is not high compared to other cancers, the patient who undergoes surgery has a better prognosis compared to patients who do not undergo surgery [28]. Patients who developed NOD before PC diagnosis have worse outcomes compared to patients with LTDM or NOD after surgical resection or those who have PC with no DM [29,30]. NOD predicts the most aggressive course of cancer and is considered as one of the strongest prognostic factors to predict the recurrence. NOD reduces the overall survival and disease-free survival in low-tumor (T; T0-T1) stage cancer; however, in one study, there was no significant difference in overall or disease-free survival compared to high T stage cancer [29]. This complex relationship was supported by the hypothesis that the loss of pancreatic parenchyma can cause NOD in aggressive types of PC. Inflammatory and pro-inflammatory factors could play a role in cell proliferation, migration, and metastasis. However, this finding is not significant and requires further investigation. Mortality after surgery within 90 days are similar in all cohorts of patients (NOD, LTDM, no DM). Patients with LTDM are prone to increased mortality due to related complications [30]. A pooled analysis of three large case-control studies found that PC with neither LTDM nor NOD was associated with short survival. However, when the results are stratified according to the stage of cancer, patients with LTDM accounted for 42% of the increased death rate in patients with resectable cancer [31].

On the other hand, another study that assessed glucose metabolism by analyzing plasma glucose and HbA1c levels in PC patients after PD showed that 36.8% of patients resolved their DM after tumor resection and 18% of patients who were not diabetic preoperatively developed DM [32]. It also explained that insulin resistance improved significantly, but insulin secretion decreased postoperatively. However, it explained that there is an improvement in glucose levels after resection in both PC and non-PC patients. This is explained by the restoration of increased glucagon-like peptide secretion and the stimulating of insulin by preventing fibrosis of the tissue by early resection. But patients undergoing 70-80% resection of the pancreas had 64.7% of the incidence of new-onset glucose impairment [33]. However, in their study, PD due to all types of pancreatic pathology was included (chronic pancreatitis, benign and malignant tumors). Patients with prior risk factors like obesity-level BMI (>23 kg/m2) and high cholesterol (>200 mg/dl) are more prone to develop glucose impairment and should be closely monitored for a longer period. Another study showed an incidence of post-surgery glucose impairment/DM of 22% within a 32-month time period [34]. However, there was a wide range in the incidence of development proposed, which was influenced by the various factors taken into account in the above study. Also, it only included patients with pancreatic ampullary cancer and excluded all other types of cancer. Therefore, more studies are required to test the changes in glucose metabolism in PC with or without DM preoperatively and about the management of impaired glucose levels after PD.

This review has some limitations. We only included studies conducted in humans. Moreover, we only reviewed studies published in English and conducted in the last five years. Perhaps, some potential good-quality papers relevant to this article might have been excluded in the study selection process. Some of the selected studies are observational, and some of them are retrospective. We only focused on the association between type 2 DM and PAC in general, and the association between other types of DM and other PC types was not really part of the scope of our study.

## Conclusions

In this paper, we reviewed the association between DM and PC in the middle-aged and elderly population (>45 years). LTDM is considered to be an independent risk factor for PC development in the elderly. NOD is a consequence and maybe the only early presenting sign of PC. There are several strategies and screening tests proposed that can be used in a stepwise manner in the detection of early-stage PC in NOD patients. A significant association was observed between DM and PC even after the treatment of PC with surgery and such patients should be monitored. However, most of the studies we reviewed were observational, and we believe that more interventional studies are required to further explore this area and to propose definitive early screening tests for PC in NOD and effective methods of postoperative management and follow-up of PC with DM.
